# Advances in Nanomaterials for Energy Conversion and Environmental Catalysis

**DOI:** 10.3390/nano14231902

**Published:** 2024-11-27

**Authors:** Jian Qi, Hui Liu

**Affiliations:** 1 State Key Laboratory of Biochemical Engineering, Institute of Process Engineering, Chinese Academy of Sciences, Beijing 100190, China; 2 State Key Laboratory of Mesoscience and Engineering, Institute of Process Engineering, Chinese Academy of Sciences, Beijing 100190, China

Not only are solutions to energy and environmental issues essential in long-term planning for the Earth’s ecological balance and sustainable economic development, they also represent an urgent issue posing a direct threat to human health. With the excessive exploitation of traditional energy and increasingly serious environmental pollution, environmental problems such as deteriorating air quality and water and soil pollution are frequently occurring. This not only exacerbates global climate change but also leads to a significant increase in health risks such as respiratory diseases, cardiovascular diseases, and cancer. Therefore, promoting energy transformation and achieving carbon peak and carbon neutrality goals have become a global shared responsibility and mission [1,2]. In this process, the design of advanced materials plays a crucial role. It is not only at the core of improving energy efficiency, reducing carbon emissions, and developing clean energy technologies, but also represents the key to improving environmental quality and protecting human health. Breakthroughs in new energy storage and conversion technologies such as lithium-ion batteries [3,4,5,6,7], sodium-ion batteries [8,9,10], potassium-ion batteries [11,12,13], zinc-ion batteries [14], lithium–sulfur batteries [15], solar cells [16], fuel cells [17,18], and all-solid-state batteries [19] are due to design and research and development work in high-performance electrode and electrolyte materials. The innovation of hydrogen production technology [20,21,22,23,24,25] and the efficient reduction of CO_2_ to produce energy through small molecules rely on innovation in efficient catalytic materials [26,27,28,29,30,31]. The degradation of pollutants [32,33,34,35] and the elimination of methane in the air depend on the design and optimization of porous catalytic materials [36,37]. In addition, research on using porous catalytic materials to treat diseases in the human microenvironment is bringing about revolutionary changes in the medical field [38]. By precisely regulating chemical reactions in the body, it provides a new treatment approach for difficult-to-treat diseases such as cancer. In summary, the importance of advanced material design in addressing energy, environmental, and health issues is self-evident, and the continuous emergence of new technologies and materials is leading us towards a greener, healthier, and more sustainable future.

This Special Issue brings together thirteen articles, including eleven research articles and two review articles, mainly focusing on the current progress and development trends in advanced materials in energy, the environment, and catalysis. The contents of the Special Issue include the following: the rate-dependent stability and electrochemical behavior of Na_3_NiZr(PO_4_)_3_ in sodium-ion batteries [39], biomass-derived carbon for lithium-ion batteries [40], PtCo/C catalysts for low-temperature fuel cells [41], high-performance photo-Fenton degradation of organic pollutants [42], carbon nanofiber anodes for superior sodium storage [43], closed pores enhancing sodium-ion energy storage [44], the effect of conductive additive on electrochemical performance [45], methane catalytic combustion under lean conditions [46], Pt/TiO_2_-Carbon photocatalysts for hydrogen production [47], multiple-interfaced nanostructures for overall water splitting [48], carbon-supported PdCu alloy for methanol electrooxidation [49], metal–organic framework nanomaterials for catalytic tumor therapy [50], and Co_3_O_4_-based composites for methane combustion [51].

This Special Issue will promote and accelerate the rational design of application-orientated advanced materials, which is of great significance in the development of new materials in energy, the environment, and catalysis, and it will also be of interest to readers in related material science fields.
